# Addition of multiple rare SNPs to known common variants improves the association between disease and gene in the Genetic Analysis Workshop 17 data

**DOI:** 10.1186/1753-6561-5-S9-S97

**Published:** 2011-11-29

**Authors:** Jenna Sykes, Lu Cheng, Wei Xu, Ming-Sound Tsao, Geoffrey Liu, Melania Pintilie

**Affiliations:** 1 Department of Biostatistics, Princess Margaret Hospital, 610 University Avenue, Toronto, ON M5G 2M9, Canada; 2 Dalla Lana School of Public Health, University of Toronto, 155 College Street, Toronto, ON M5T 3M7, Canada; 3 Laboratory of Medicine and Pathobiology, Princess Margaret Hospital, 610 University Avenue, Toronto, ON M5G 2M9, Canada; 4 Division of Molecular Genomics, Princess Margaret Hospital, 610 University Avenue, Toronto, ON M5G 2M9, Canada

## Abstract

The upcoming release of new whole-genome genotyping technologies will shed new light on whether there is an associative effect of previously immeasurable rare variants on incidence of disease. For Genetic Analysis Workshop 17, our team focused on a statistical method to detect associations between gene-based multiple rare variants and disease status. We added a combination of rare SNPs to a common variant shown to have an influence on disease status. This method provides us with an enhanced ability to detect the effect of these rare variants, which, modeled alone, would normally be undetectable. Adjusting for significant clinical parameters, several genes were found to have multiple rare variants that were significantly associated with disease outcome.

## Background

Recent technological advances have made querying the importance of genetic factors on the occurrence of disease severity possible. Hundreds of published studies have acknowledged associations between certain genes and various medical conditions. Newer advances in genotyping technology have allowed researchers to determine even more precisely which genetic base pair may be a marker for the mutation responsible for causing a disease by looking at single-nucleotide polymorphisms (SNPs). SNPs are DNA sequence variations that occur when a single nucleotide (A, T, C, or G) in the genome is altered. Each individual has many SNPs that together create the unique human DNA pattern [1]. These base differences usually have a minor allele frequency (MAF) of 1% or more; SNPs with MAFs less than 1% are known as rare [2]. Previously, because of the popular common disease/common variant hypothesis, which assumes that common diseases are caused by common variants with small to modest effects [3], and because of the lack of proper technology to accurately genotype rare variants, most association studies have focused on common variants. The near complete 1000 Genomes Project will allow for more accurate genotyping of the so-called rare variants and, as a result, for consideration of rare variants as possible causes of disease [4].

A change in thought has occurred to increase the importance of rare variants in disease susceptibility [5]. Although several common SNPs have shown significant associations with diseases, these effect sizes have always been small, contributing to the idea that there must be some causal factor in the previously undiscovered rare variants [5]. Several known genetic diseases, such as schizophrenia and type 2 diabetes, have turned up only a few links in the form of the common variants, and it is now thought that common variants could be picking up a diluted signal that is instead caused by neighboring rare variants [5]. Few statistical methods exist for analyzing the role of rare variants, with most methods resulting in low power [3], and it is imperative to develop new methods to analyze these data. Because the Genetic Analysis Workshop 17 (GAW17) data set is dominated by rare variants (about 74%), the goal of this study is to investigate the potential for combinations of rare variants to strengthen the association between common variants and disease.

## Methods

The GAW17 data set consists of 24,487 SNPs on 22 chromosomes for 697 unrelated individuals. Thirty percent of the individuals are known to be affected with the disease, and individual quantitative and binary disease traits, Age, Sex, and Smoking status were simulated 200 times. The underlying simulation model is presented by Almasy et al. [6]. We had no knowledge of the genes simulated to be associated with disease outcome when developing and testing our method.

We chose significant clinical parameters by fitting a multivariate logistic regression model with all the possible covariates (Age, Sex, Smoking status, and Ethnicity) and performing backwards selection. Significance was determined by calculating the 95% percentile intervals based on the 200 replicates and choosing only those covariates for which the percentile interval did not include 0.

We first tested for Hardy-Weinberg equilibrium (HWE) in both affected and unaffected populations over all 200 phenotype replicates [7]. An adjusted *p*-value of 10^−5^ was used to correct for multiple testing in light of the fact that many of the SNPs are correlated. Those SNPs that failed the HWE test in both subpopulations in at least 95% of the replicates were eliminated from further analysis because these SNPs were thought to be privy to genotyping errors.

Because the frequency of each of the rare variants in this data set is so low (40% of the SNPs have only a single copy of the minor allele out of the 697 observations), attempting to model the relationship between each rare SNP and the disease outcome is not feasible. Even attempting to combine all the rare SNPs within a gene would not be possible because few genes have a large number of rare SNPs (Table 1). Under these conditions, the models would fail to converge in many of the phenotypes. Therefore we decided to test combinations of multiple rare variants with one common variant in a gene. Our interest lies in identifying groups of rare SNPs that will better predict the disease when added to the common SNP than in simply identifying the common SNP alone.

**Table 1:** Breakdown of number of rare SNPs per gene

Number of rare SNPs	Number of genes
None	691
Between 1 and 5	1,601
Between 6 and 10	338
Between 11 and 50	502
Between 51 and 100	29
More than 101	6

For each gene *g*, we consider common SNPs *c_j_* (*j* = 1, …, *n_g_c__*) and rare SNPs *r_s_* (*s* = 1, …, *n_g_r__*), where *n_g_c__* and *n_g_r__* are the number of common and rare SNPs on gene *g*, respectively. For all SNPs, we assume a dominant model in which a SNP is coded 1 when a minor allele is present and 0 otherwise. Because of the low frequency of rare SNPs, we thought that the dominant model would provide the best power.

For individual *i*, *i* = 1, …, 697, we define disease status as:(1)

For each *c_j_* on gene *g*, we fit the following multivariate logistic regression model on phenotype *k* (*k* = 1, …, 200):(2)

where *P* = *P*(**Y** = 1) and **1**_c_j_ >0_ is a binary indicator variable representing the presence of the minor allele in common SNP c*_j_.*. The 200 coefficients *β*_1,1_, …, *β*_1,200_ are recorded.

We create a new indicator variable *z* to measure the presence of rare variants within a gene:(3)

By narrowing the search to only those common variants that show reproducibility over the 200 replicates at the 0.1 significance level (which would imply more stable coefficient estimates), we then fit a new multivariate logistic regression model with a binary indicator variable that represents the presence or absence of any minor allele within the gene:(4)

We use the binary approach to increase the power to detect an association resulting from the low frequency of the minor alleles. We then compare the 200 coefficients *γ*_1,1_, …, *γ*_1,200_ by means of a one-sided paired *t* test to *β*_1,1_, …, *β*_1,200_ to ascertain whether there is a consistent departure from the null hypothesis that:(5)

If *p* < 0.05, then adding the rare variants to the common variant significantly increases the signal of the effect of the gene on disease. Therefore these rare variants must be associated with the disease.

If no associations are found, we remove one rare SNP from the definition of Eq. (3) and recalculate the coefficients from Eq. (4) as before. This method is used to determine whether or not no association was detected because of too much noise resulting from the addition of too many rare variants. This method can be generalized through an iterative process by removing one rare SNP at a time until only a single rare SNP remains.

## Results

**Figure 1:**
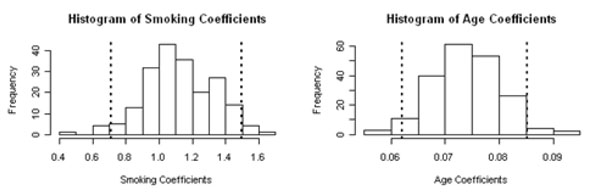
Plot of the coefficients over all 200 phenotypes shows that Smoking status and Age have significant effects on disease

The initial set of 24,487 SNPs was reduced to 24,211 because 276 SNPs failed the HWE assumption. Ethnicity was categorized into three dummy variables representing individuals of African, Asian, and European descent. The covariates Age and Smoking status were established as the only clinical parameters for this data set (Figure 1). Any associations between SNPs and disease status were adjusted for these two covariates.

After HWE elimination, we were left with 3,167 genes over the 22 chromosomes. Of these 3,167 genes, 1,718 did not have any common variants or had less than two rare variants and so were excluded from the analysis because this is our primary interest. To eliminate the possibility of adding too much noise by creating a combination of many rare SNPs, we further restricted the analysis to those genes that contained fewer than 16 rare SNPs. Thus we were left with 829 genes to explore.

**Figure 2:**
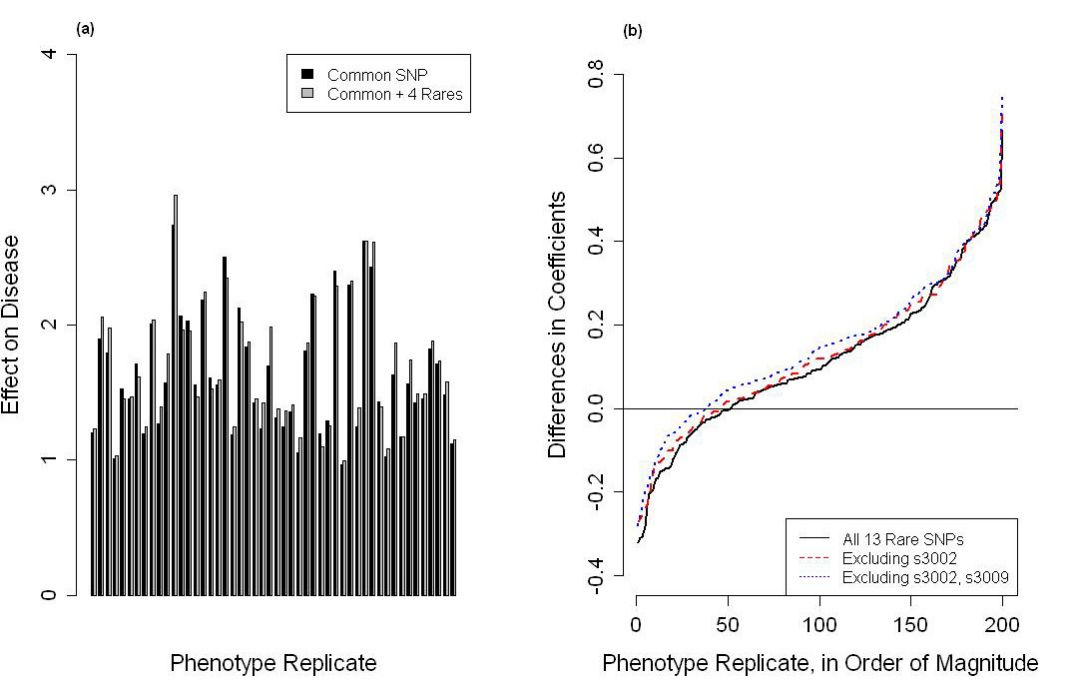
**Addition of rare SNPs increases signal for detection.** (a) For gene *MAP3K6*, a combination of four rare SNPs and one common SNP increases the signal. (b) For gene *BRCA1* we show the coefficient differences between various rare and common SNP combinations and a common SNP.

Our results show that adding multiple rare variants to common SNPs already associated with disease at the 0.1 significance level can greatly improve the ability to detect causes of disease (Table 2). We calculated *p*-values from a one-sided paired *t* test to compare the 200 *β* coefficients to the 200 *γ* coefficients and used a *p*-value of 0.001 to determine significance [8]. For several of the genes, the signal of association became even stronger with the removal of one or two rare SNPs (Figure 2). In some cases, we discovered that larger combinations of rare SNPs were actually more significant, indicating that an optimal combination of rare and common SNPs could be found with this method (Table 2).

**Table 2:** Adding a combination of rare SNPs to a common SNP significantly increases the signal of association with disease

Gene	Common SNP	Number of rare SNPs	*p*-value	SNP(s) removed	*p*-value
*MAP3K6*	C1S1886	4	2.13 × 10^−8^	C1S18877	2.13 × 10^−8^
*PTK2B*	C8S911	9	1.08 × 10^−7^	C8S936	2.9 × 10^−3^
				C8S900	5.12 × 10^−9^
				C8S900, C8S908	8.93 × 10^−10^
*ETV6*	C12S860	2	5.53 × 10^−7^	C12S863	3 × 10^−4^
				C12S861	6 × 10^−4^
*BRCA1*	C17S2996	13	1.54 × 10^−16^	C17S3009	1.97 × 10^−21^
				C17S3002, C17S3009	1.01 × 10^−25^
*BRCA1*	C17S3006	13	4.45 × 10^−11^	C17S3002	2.88 × 10^−14^
*BRCA1*	C17S3010	13	7.27 × 10^−10^	C17S3002	6.73 × 10^−13^
*BRCA1*	C17S3014	13	5.33 × 10^−9^	C17S3002	5.04 × 10^−12^
*BRCA1*	C17S3016	13	1.31 × 10^−10^	C17S3009	8.75 × 10^−15^
*LOC645118*	C19S2844	1	NS	C19S2846	9 × 10^−4^
*TNK1*	C17S511	3	NS	C17S515, C17S521	4.78 × 10^−5^
*VNN1*	C6S5380	4	NS	C6S5377, C6S5379	1.5 × 10^−4^

Removing one or two rare SNPs can make this association even stronger. NS, not significant at the 0.001 significance level.

When one or two rare SNPs were removed from the definition of Eq. (3), some genes that had not been identified by our first pass displayed an increased effect on disease (Table 2, last three rows). This suggests that adding the combination of rare SNPs to a common SNP adds information to the model and helps to better explain the relationship between gene and disease.

## Discussion and conclusions

By taking advantage of all 200 phenotype replicates, we were able to simulate a posterior distribution for the underlying true relationship between genes and disease status, thereby inherently validating our method. When working with real data, investigators will not be able to use the replications to calculate *p*-values. Therefore we can apply the sample randomization technique outlined by Guo et al. [9]. This method has the following steps: (1) Calculate the coefficient for each common variant in each gene from a logistic regression model; (2) shuffle the common SNPs across the genome to generate a permuted data set; (3) calculate the coefficient from a logistic regression between common variant and disease; (4) repeat steps 2 and 3 1,000 times to obtain a null distribution of coefficients; and (5) determine which common variants are significant (at *α* = 0.1) by calculating the percentage of coefficients from the null distribution that are greater than the observed coefficient. This percentage is our *p*-value. Finally, adding all the rare SNPs to the common variant, we repeat steps 1–5 to determine which rare SNPs significantly improve the association from the common variant alone.

Although our study focused on binary disease outcome, our method can also be applied to continuous or time-to-event outcomes. The dominant model assumption for the SNPs could also be adjusted to use additive or recessive models. Our method improves on the collapsing method introduced by Li and Leal [3] by separately considering common variants shown to have disease influence and by adjusting for other factors.

In the interest of time and computational abilities, we limited our analysis to those genes with less than 16 rare SNPs. Important associations may occur in genes with greater than 16 SNPs. In the future it may also be of interest to consider separately those SNPs that are synonymous and nonsynonymous or to include rare SNPs that fall just outside a gene in a larger genomic region in, say, a pathway-based analysis. Our analysis was stopped before considering a maximum removal of two rare SNPs from the combination of all rare SNPs in one gene. A more exhaustive search could uncover new relationships. Our intention was to conduct a proof of principle analysis to exhibit the merits of this method in finding rare SNPs associated with disease.

After the GAW17 conference, we compared the performance of our method to the simulated answers. For the correctly identified gene *PTK2B*, removal of simulated SNP C8S900 actually improved the disease association. This could be a result of high correlation with the other simulated SNPs for that gene. Table 3 shows that our method detected a large number of false positives and yielded a sensitivity of only 8.3%. However, our method had quite a high specificity rate of 98.5%. It must be noted that underlying correlation could create hidden relationships not specified in the simulated model.

**Table 3:** Gene-based comparison of our method with simulated answers

	Our method		Total
		
Answers	Selected	Not selected	
Simulated	2	22	24
Not simulated	9	793	805
Total	11	815	829

## Competing interests

The authors declare that there are no competing interests.

## Authors’ contributions

JS performed and interpreted the statistical analysis and drafted the manuscript. WX, GL and LC acted as advisors on the design of the statistical analysis. MP participated in the planning of the analysis and structuring of the paper. All authors read and approved the final manuscript.
